# Mitochondria: the beating heart of the eukaryotic cell

**DOI:** 10.1002/2211-5463.13884

**Published:** 2024-10-04

**Authors:** Johannes M. Herrmann

**Affiliations:** ^1^ Cell Biology University of Kaiserslautern, RPTU Kaiserslautern Germany

## Abstract

Mitochondria are essential organelles of eukaryotic cells. They consist of hundreds of proteins, which are synthesized in the cytosol and imported into mitochondria via different targeting routes. In addition, a small number of proteins are encoded by the organellar genome and synthesized by mitochondrial ribosomes. In this ‘In the Limelight’ special issue of *FEBS Open Bio*, five review articles describe these different biogenesis routes of mitochondrial proteins and provide a comprehensive overview of the structures and mechanisms by which mitochondrial proteins are synthesized and transported to their respective location within the organelle. These reviews, written by leading experts, provide a general overview, but also highlight current developments in the field of mitochondrial biogenesis.

**Abbreviations**
IMS: intermembrane space
SAM: sorting and assembly machinery
TIM: translocase of the inner mitochondrial membrane
TOM: translocase of the outer mitochondrial membrane

Mitochondria are often referred to as the powerhouse of the cell. This emphasizes their importance in energy metabolism which is impressive: in a single human individual, mitochondria produce about 60 kg of ATP per day by aerobic respiration (net production) fueling all kinds of reactions. However, the narrative of the powerhouse falls short with respect to the many other crucial functions of mitochondria. For example, mitochondria mediate the elementary reactions of the biogenesis of iron–sulfur clusters, making them essential even in non‐respiring cell types and organisms [1]. Moreover, mitochondrial enzymes synthesize heme, ubiquinone, and several amino acids and lipids. Furthermore, mitochondria are important for the calcium, metal, and redox homeostasis, execute apoptosis, are hubs in antiviral signaling pathways, and are critical for aging [2]. The central, essential role of mitochondria resembles that of the heart which ‘energizes’ our body. In that picture, the nucleus might be similar to the brain, commanding cellular decisions. The brain and heart are the life‐defining organs, and the nucleus and mitochondria are the vital cellular organelles.

Mitochondria consist of about 1000 (yeast) to 1500 (humans) proteins to carry out all these different functions [3, 4]. Only a very small number of proteins are encoded in the mitochondrial genome: 8 in yeast and 13 in humans. All other mitochondrial proteins are synthesized on cytosolic ribosomes and imported, using the TOM complex as the common entry gate (Fig. 1). Several import pathways sort the different proteins to their specific intramitochondrial location and ensure the correct topology of membrane proteins. This ‘In the Limelight’ special issue of *FEBS Open Bio* comprises five articles, which illustrate and discuss different aspects of these mitochondrial protein biogenesis pathways.

**Fig. 1:**
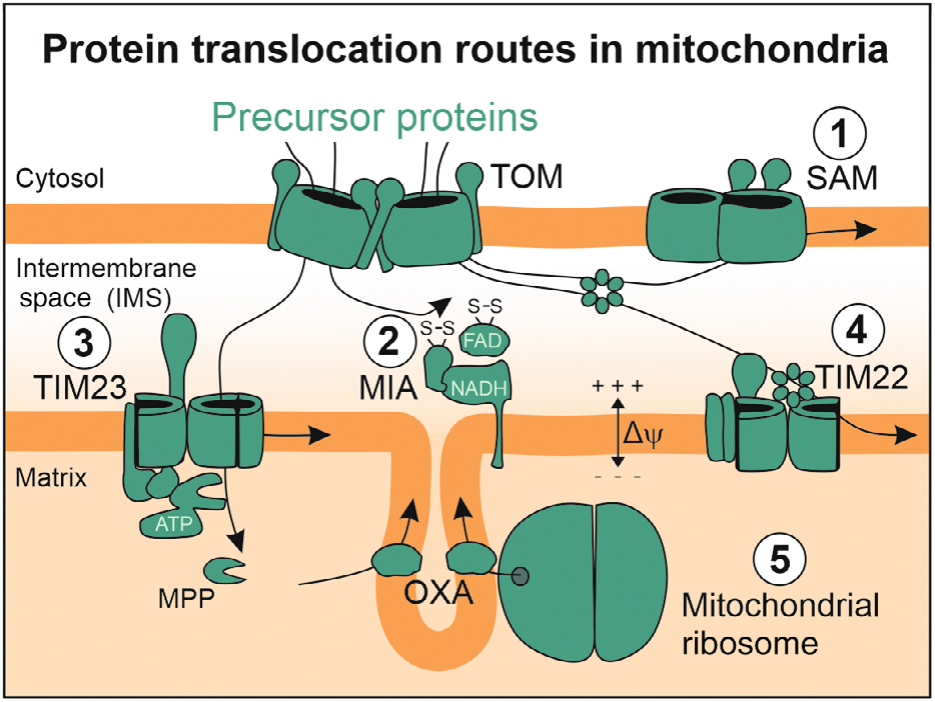
Mitochondrial proteins reach their destination in mitochondria on different targeting routes. The TOM complex serves as an entrance gate for almost all nuclear‐encoded mitochondrial proteins. After translocation across the outer membrane, proteins can embark on the SAM pathway to get inserted into the outer membrane (Pathway 1), on the MIA pathway which introduces disulfide bonds into its substrates (Pathway 2), on the presequence pathway to traverse the inner membrane (Pathway 3) or on the TIM22 pathway which inserts carrier proteins into the inner membrane (Pathway 4). Translation products of the mitochondrial ribosome are inserted into the inner membrane in a co‐translational manner by the OXA pathway (Pathway 5). These five different pathways are introduced and discussed in this ‘In the Limelight’ special issue of *FEBS Open Bio*, emphasizing the current development of the field which often were inspired by recent cryo‐electron microscopy structures.

The outer membrane of mitochondria contains pore‐forming beta‐barrel proteins, which facilitate the diffusion of molecules of up to about 3000 Dalton across the outer membrane. Beta‐barrel proteins are also found in the outer membranes of bacteria and chloroplasts, revealing the common evolutionary origin. Owing to this bacterial descent, mitochondrial beta‐barrel proteins are integrated from the ‘inside’, that is, from the intermembrane space (IMS). After being translocated through the TOM complex, precursors of beta‐barrel proteins are stitched into the outer membrane in an export‐like process by the sorting and assembly machinery (SAM) complex. Cryo‐electron microscopy structures of the SAM complex, in some cases with associated translocation intermediates, have recently revealed fascinating details of this machinery [5, 6, 7, 8]. Nils Wiedemann, Nikolaus Pfanner, and coworkers provide a comprehensive overview of the structure and function of the beta‐barrel assembly machinery [9].

During the evolution of eukaryotic cells, the IMS of mitochondria was derived from the periplasmic space of the endosymbiont. Presumably owing to the same evolutionary descent, soluble proteins of the bacterial periplasm and of the IMS reach their three‐dimensional structure by oxidative protein folding. The review by Christine Zarges and Jan Riemer describes the enzymes of the human IMS, which insert disulfide bonds into newly imported proteins [10]. The two major enzymes, the FAD‐bound sulfhydryl oxidase ALR/Erv1 and oxidoreductase CHCHD4/Mia40, are conserved from yeast to human. However, in humans, Mia40 is tethered to the inner membrane by the NADH dehydrogenase AIF adding an interesting regulatory component to the human mitochondrial disulfide relay.

Most mitochondrial proteins reside in the matrix. These proteins are synthesized in the cytosol with aminoterminal presequences to direct them into mitochondria. The presequence translocase of the inner membrane (also known as the TIM23 complex) facilitates their translocation across the inner membrane. Recent structures of the TIM23 complex of yeast showed that Tim17 represents the protein‐conducting essential subunit contradicting previous assumptions [11, 12]. Tim17 forms a half‐channel that is partially exposed to the lipids of the inner membrane and uses negatively charged residues to promote the passage of the positively charged presequences into the matrix [13]. In this ‘In the Limelight’ special issue of *FEBS Open Bio*, Agnieszka Chacinska and coworkers show models of the human TIM23 translocase and compare its structure to that of yeast cells [14].

The biogenesis of inner membrane proteins is particularly complex as they can use three distinct translocation routes: Proteins can be arrested in the TIM23 complex and laterally inserted into the lipid bilayer, they can insert from the matrix with the help of the Oxa1 insertase protein or, finally, they can employ the TIM22 complex which inserts proteins from the IMS into the inner membrane. The fourth article of this collection describes the molecular details of these three insertion mechanisms [15].

Mitochondria make a big effort to express the small number of mitochondrially encoded proteins. For the synthesis of these 8 or 13 polypeptides, about 150 different nuclear‐encoded proteins are necessary, including about 80 proteins of the mitochondrial ribosome. In their article, Antonio Barrientos and coworkers explain how the mitochondrial ribosome is assembled, putting particular emphasis on thiol‐based redox processes [16]. The mitochondrial ribosome was one of the first complexes analyzed by cryo‐electron microscopy and recent studies even showed the architectures of assembly intermediates [17]. This is again a wonderful example documenting the powerful combination of structural biology with biochemistry to reveal the mechanisms underlying mitochondrial protein biogenesis and thus the molecular processes by which the ‘beating heart of the cell’ is put together.

I thank all authors for their exciting contributions to this ‘In the Limelight’ special issue of *FEBS Open Bio*.

## Conflict of interest

The authors declare no conflict of interest.

## Author contributions

JMH wrote the article.
